# Prospective assessment of two-gene urinary test with multiparametric magnetic resonance imaging of the prostate for men undergoing primary prostate biopsy

**DOI:** 10.1007/s00345-020-03359-w

**Published:** 2020-07-17

**Authors:** Gian Maria Busetto, Francesco Del Giudice, Martina Maggi, Ferdinando De Marco, Angelo Porreca, Isabella Sperduti, Fabio Massimo Magliocca, Stefano Salciccia, Benjamin I. Chung, Ettore De Berardinis, Alessandro Sciarra

**Affiliations:** 1 Department of Urology, Sapienza Rome University, Policlinico Umberto I, Viale del Policlinico 155, 00161 Rome, Italy; 2 I.N.I Istituto Neurotraumatologico Italiano-Urology Division-Grottaferrata, Grottaferrata, Rome, Italy; 3 Department of Urology, Policlinico Abano Terme, Abano Terme, Italy; 4 Biostatistical Unit-IRCCS, Regina Elena National Cancer Institute, Rome, Italy; 5 Department of Radiological, Oncological and Anatomopathological Sciences, Sapienza Rome University, Policlinico Umberto I, Rome, Italy; 6 Department of Urology, Stanford University Medical Center, Stanford, CA USA

**Keywords:** mpMRI, Prostate biopsy, Prostate neoplasm, PSA, Urinary biomarker

## Abstract

**Purpose:**

To evaluate the diagnostic accuracy of SelectMDx and its association with multiparametric magnetic resonance (mpMRI) in predicting prostate cancer (PCa) and clinically significant PCa (csPCa) on prostate biopsies among men scheduled for initial prostate biopsy.

**Methods:**

In this single-center prospective study, 52 men scheduled for initial prostate biopsy, based on elevated total PSA level (> 3 ng/ml) or abnormal digital rectal examination, were consecutively included. All subjects underwent SelectMDx, PSA determination and mpMRI.

**Results:**

SelectMDx score was positive in 94.1 and 100% of PCa and csPCa, respectively, and in only 8.6% of negative cases at biopsy. The probability for a csPCa at the SelectMDx score was significantly (*p* = 0.002) higher in csPCa (median value 52.0%) than in all PCa (median value 30.0%). SelectMDx showed slightly lower sensitivity (94.1 versus 100.0%) but higher specificity (91.4%) than total PSA (17.1%), and the same sensitivity but higher specificity than mpMRI (80.0%) in predicting PCa at biopsy. The association of SelectMDx plus mpMRI rather than PSA density (PSAD) plus mpMRI showed higher specificity (both 91.4%) compared to the association of PSA plus mpMRI (85.7%). In terms of csPCa predictive value, SelectMDx showed higher specificity (73.3%) than PSA (13.3%) and mpMRI (64.4%); as for the association of SelectMDx plus mpMRI (75.6%) versus PSA plus mpMRI (68.9%), the association of PSAD plus mpMRI showed the highest specificity (80.0%).

**Conclusion:**

Our results of SelectMDx can be confirmed as significant but their impact on clinical practice together with a cost-effectiveness evaluation should be investigated in a larger prospective multicenter analysis.

**Electronic supplementary material:**

The online version of this article (10.1007/s00345-020-03359-w) contains supplementary material, which is available to authorized users.

## Introduction

Prostate cancer (PCa) is the most commonly diagnosed cancer and the second leading cause of cancer related deaths in men. Furthermore, PCa and subsequent treatments have a high impact on both functional and psychological status, significantly affecting patients’ Quality of Life (QoL) [1].

Over the years PCa screening has been one of the most controversial topics in urology, and currently there is insufficient evidence to justify the introduction of population-based PCa screening programs based on prostate-specific antigen (PSA) measurement.

According to the European Association guidelines, an individualized risk-adapted strategy for early detection of PCa might be offered to a well-informed man with at least 10–15 years of life expectancy [2].

The current standard method of diagnosing PCa is transrectal ultrasound (TRUS)-guided prostate biopsy, which is mainly performed on the basis of PSA level [2]. Major issues associated with PSA testing are: 1—the absence of a cut-off value associated with high specificity and sensitivity, and 2—PSA is organ but not cancer-specific (it may be elevated in benign prostatic hyperplasia (BPH), inflammation and other non-malignant conditions). According to these considerations PCa screening using a PSA-based threshold as the sole indication for prostate biopsy lacks specificity, resulting in large numbers of unnecessary biopsies and, at the same time, in missing cancer diagnoses in men with PSA levels below the cut-off value [3].

In order to add sensitivity and specificity on PSA and avoid unnecessary biopsies, over the years, many tools have been developed. Multiparametric magnetic resonance imaging (mpMRI) in PCa has demonstrated better outcomes in terms of increased cancer diagnosis via targeted biopsy, decreased diagnosis of indolent PCa, and improved risk stratification [4, 5]. Currently there is the evidence to recommend the use of mpMRI not only for re-biopsies but also in biopsy-naïve men [2].

In the last years, several risk calculators, which combine PSA and other risk factors (e.g., age, family history and ethnicity), have been developed to aid urologists in determining patients’ individual risk for both PCa and clinically significant PCa (csPCa). Despite over a hundred predictive models are available online, currently their clinical benefit and side-effects related to over-diagnosis need to be proven.

Additional serum assays testing a panel of kallikreins have been approved: Prostate Health Index (PHI) test (considering free and total PSA and the [–2]pro-PSA isoform [p2PSA]) and the four kallikrein (4 K) score (considering free, intact and total PSA and kallikrein-like peptidase 2 [hK2]) showed similar results in reducing unnecessary prostate biopsies [6].

Urine-based tests, such as Progensa, measuring mRNA biomarker [prostate cancer gene 3 (PCA3)] in urine sediments, are also available [7, 8]. Thus, all these issues suggest that there is still a real need for urologists to base clinical decisions on more efficient tools able to reduce over-diagnosis of potentially indolent PCa and improve detection of csPCa.

SelectMDx is a post-digital rectal examination (DRE) urine methylation assay available in clinical practice to improve patient selection for initial prostate biopsy [9]. Leyter et al. selected eight biomarkers according to a quantitative polymerase chain reaction (PCR) analysis of urinary sediments [10]. A validated urinary 3 genes panel (HOXC6, TDRD1 and DLX1) showed high accuracy (AUC = 0.77) for the detection of csPCa compared with PSA and PCA3 test (AUC = 0.72 and 0.68, respectively) [10]. SelectMDx is a novel biomarker-based risk score assessing urinary HOXC6 and DLX1 mRNA expression combined with tradition clinical risk factors. This risk score reached an AUC of 0.86 in a validated cohort and could therefore reduce the number of unnecessary biopsies [11, 12]. However, there are too limited data to implement these markers into routine screening programs.

Aim of this prospective single-center study was to evaluate the diagnostic accuracy of SelectMDx and its association with mpMRI in predicting PCa and csPCa on prostate biopsies among men scheduled for initial prostate biopsy.

## Materials and methods

### Study population

This is a single-center prospective study conducted in line with European Urology and Good Clinical Practice guidelines, with ethical principles laid down in the latest version of the Declaration of Helsinki. Informed consent was obtained from all individual participants included in the study. Men who were scheduled for initial prostate biopsy, based on elevated total PSA level (> 3 ng/ml confirmed) or abnormal digital rectal examination (DRE) were consecutively included between March 2018 and September 2019. Exclusion criteria were a history of PCa or other neoplasm under active treatments, medical therapies known to affect PSA and the prostate gland, invasive treatments for BPH, prior prostatic biopsy. Table 1 shows patients’ characteristics.

**Table 1:** Patients’ characteristics (number, %, mean ± SD, median, range)

Parameter	Value
Number of cases, *n*	52
Age (years)	
Mean ± SD	64 ± 8.7
Median	67
Range	44–79
Prostate volume (ml)	
Mean ± SD	47.6 ± 21.4
Median	47.6
Range	21–95
Total PSA (ng/ml)	
Mean ± SD	6.8 ± 3.9
Median	5.9
Range	1.0–19.9
PSAD (ng/ml/ml)	
Mean ± SD	0.16 ± 0.11
Median	0.14
Range	0.02–0.53
DRE suspicious, *n* (%)	
Yes	10 (19.2)
No	42 (80.8)
Family history, *n* (%)	
Yes	10 (19.2)
No	42 (80.8)
SelectMDx score, *n* (%)	
Negative	33 (63.5)
Positive	19 (36.5)
mpMRI PI-RADS score, *n* (%)	
PI-RADS 1–2	29 (55.7)
PI-RADS 3	11 (21.2)
PI-RADS 4–5	12 (23.1)
SelectMDx score and mpMRI PI-RADS score, *n* (%)	
SelectMDx positive, mpMRI positive	18 (34.7)
SelectMDx negative, mpMRI negative	28 (53.8)
SelectMDx positive, mpMRI negative	1 (1.9)
SelectMDx negative, mpMRI positive	5 (9.6)
PSA and mpMRI PI-RADS score, *n* (%)	
PSA ≥ 3 ng/ml, mpMRI positive	21 (40.4)
PSA < 3 ng/ml, mpMRI negative	2 (3.8)
PSA ≥ 3 ng/ml, mpMRI negative	27 (52.0)
PSA < 3 ng/ml, mpMRI positive	2 (3.8)
PCa at biopsy, *n* (%)	17 (32.7)
csPCa at biopsy, *n* (%)	7 (13.5)

*n* number, *SD* standard deviation, *PSA* prostate-specific antigen, *PSAD* PSA density, *DRE* digital rectal examination, *mpMRI* multiparametric magnetic resonance imaging, *PI-RADS* prostate imaging reporting and data system, *PCa* prostate cancer, *csPCa* clinically significant PCa

### Methods

Patients’ characteristics and clinical tests results were collected.

#### mpMRI

All subjects included into the analysis were submitted to a mpMRI using a 3 T MR scanner (GE Discovery MR750). A combination of T2-weighted (T2W) images, diffusion-weighted imaging (DWI) and dynamic contrast-enhanced (DCE) studies were used as functional techniques, and the PI-RADS version 2 (v2) was used for grading the lesions with a final grade from 1 to 5 [13]. All mpMRI were performed before prostate biopsy and analyzed by expert radiologists blinded to urine test scores and biopsy outcomes.

#### SelectMDx sampling

First-voided urine samples (approximately 30 ml) were collected after a standardized DRE consisting of three strokes per lobe [7]. Samples were shipped at room temperature to a central laboratory and stored at − 80 °C. The SelectMDx score (MDx Health) is a combination of expression levels from HOXC6 and DLX1 and clinical risk factors (age, DRE, PSA, PSA density, family history, prior negative biopsies) in a logistic regression model [11]. At now, no cut-off point is provided by the test as in previous experiences [12] and the results of the test are given as positive (with the percentage of probability for PCa and csPCa) or negative in the suspicious of PCa.

#### Prostatic biopsy

All subjects were submitted to a transrectal ultrasound (TRUS)-guided prostate biopsy performed in our clinic by the same radiologist with more than 20 years of experience. In all cases 12 random systematic cores were obtained. In cases of PI-RADS score 3–5 at mpMRI, additional targeted samples (2 cores per lesion) were obtained using an imaging fusion technique (Urostation, Koelis). All samples were evaluated in our clinic by a more than 10 years’ experience genitourinary pathologist. Histological grading was assessed according to the Gleason grading system and Gleason Grade Groups (International Society of Urological Pathologist ISUP 2014) [14]. A csPCa was defined as ISUP score ≥ 2 (Gleason score ≥ 7) [2].

#### Statistical analysis

Descriptive statistics were used to describe patients’ characteristics. The association between variables was tested by the Pearson Chi Square test or the Fisher’s Exact test. The comparisons among groups were performed by Mann–Whitney *U* test or Kruskal–Wallis non parametric test, when appropriate. Performance characteristics (sensitivity, specificity, areas under the curves (AUC)) were evaluated by computing receiver operating characteristic (ROC) curves. We used decision curve analysis (DCA) to compare the clinical utility of using each tool alone and their association. Fit-splines curves analysis was used to evaluate the prediction of SelectMDx score for the detection of PCa and csPCa. The SelectMDx findings were categorized as positive (with the percentage of probability for PCa and csPCa) or negative for the suspicious of PCa by manufacturers’ report. The mpMRI results were considered positive if reported as PI-RADS 3–5, and negative if PI-RADS 1–2. To evaluate the performance of using both tests together, positive cases were those with positive biomarker and positive mpMRI (concordant cases). Finally, to evaluate discordant cases we determined the number of avoided biopsies and missed PCa by SelectMDx results and PSA density (PSAD) values. Cut-off levels were set at > 3 ng/ml for PSA, and ≥ 0.15 ng/mL/mL for PSAD [15, 16]. *P* values < 0.05 were considered significant. The SPSS (21.0) and MedCalc (14.10.2) statistical programs were used for all analyses.

## Results

### Study population

**Fig. 1:**
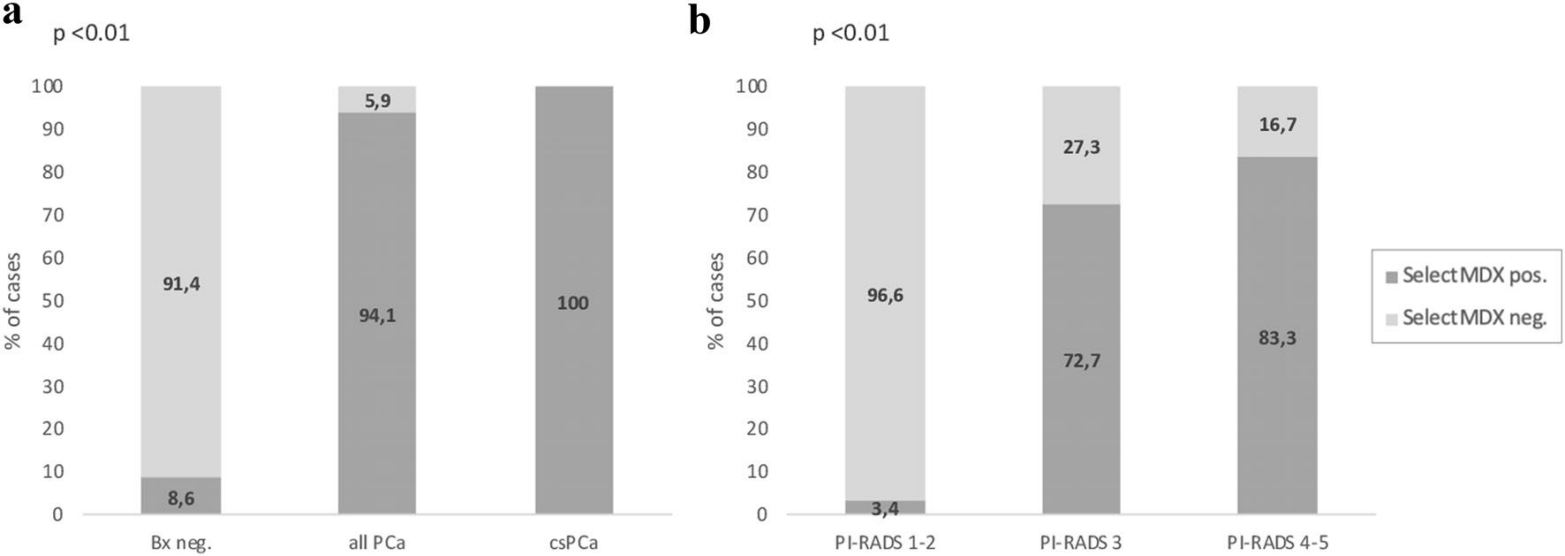
**a** SelectMDx positive and negative results according to histologic diagnosis for PCa and csPCa at biopsy; **b** SelectMDx positive results according to PI-RADS score at mpMRI

**Fig. 2:**
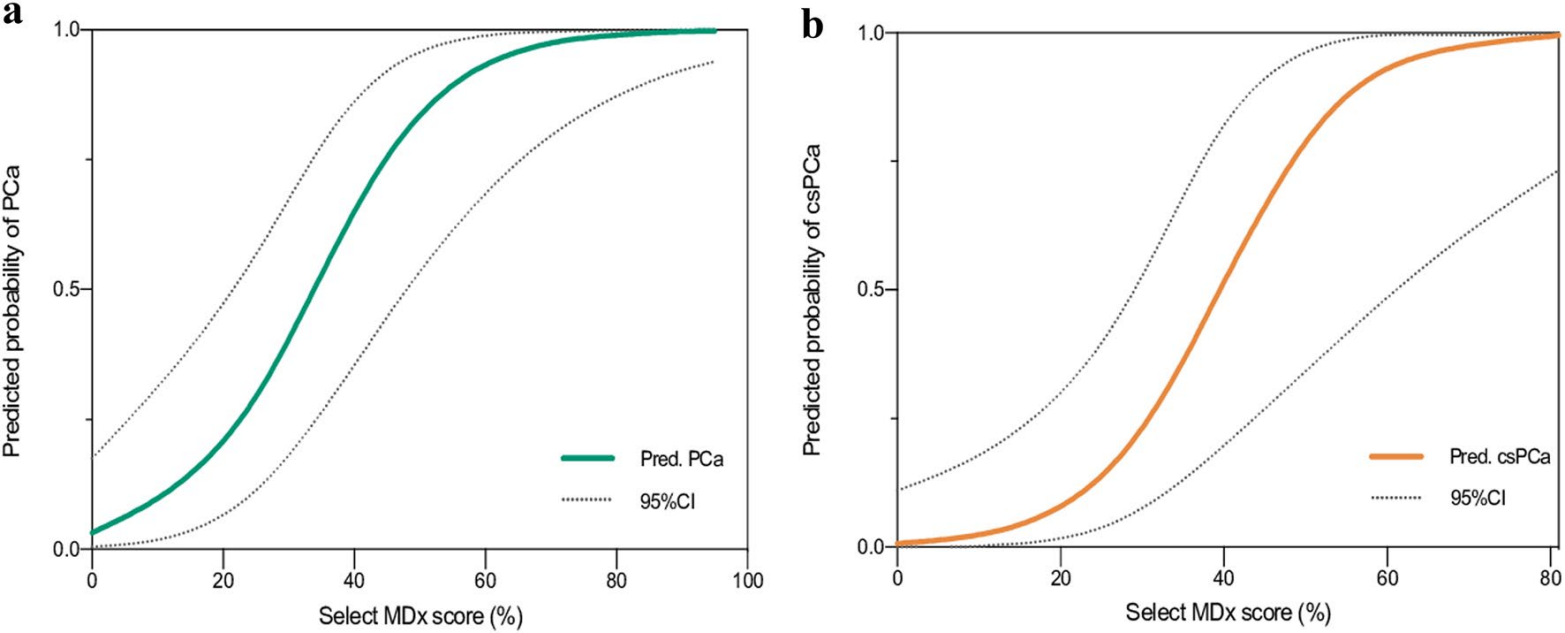
Fit-spline curves depicting predicted probability of PCa (**a**) and csPCa (**b**) according to the values of Select MDx score percentages (%)

Fifty-two consecutive subjects were included in our prospective analysis (Table 1) and completed all the examinations. A positive SelectMDx score was found in 19 cases (36.5%) and a PI-RADS score ≥ 3 in 23 cases (44.3%). SelectMDx and mpMRI were concordant in 88.5% of cases while discordant in 11.5%. At biopsy a PCa was histologically detected in 17 (32.7%) patients and 7 (13.5%) were csPCa (Table 1). Stratification of subjects on the basis of pathologic results at biopsy (PCa negative, all PCa, csPCa) is reported in Table 2. Total PSA levels significantly (*p* = 0.018) differed among the three groups (Table 2 and Supplementary Figure 1a). SelectMDx score was positive in 94.1 and 100% of PCa and csPCa, respectively, and in only 8.6% of cases with no PCa at biopsy (Table 2 and Fig. 1a) (*p* = 0.002). The probability for a csPCa at the SelectMDx score was significantly (*p* = 0.002) higher in csPCa (median value 52.0%) than in all PCa cases (median value 30.0%) (Table 2) at biopsy. The prediction of SelectMDx score (%) performance alone for the detection of PCa and csPCa is graphically displayed by fit-splines curves analysis (Fig. 2a, b, respectively).

**Table 2:** Stratification of patients’ characteristics on the basis of prostatic biopsy results (number, %, mean ± SD, median, range)

Parameter	Negative PCa	All PCa	CsPCa	*p* value
Number of cases, *n* (%)	35 (67.3)	17 (32.7)	7 (13.5)	
Age (years)				
Mean ± SD	64 ± 7.5	66 ± 10.3	62 ± 9.4	0.964
Median	64	69	66	
Range	45–74	44–79	44–70	
Total PSA (ng/ml)				
Mean ± SD	5.3 ± 2.5	9.9 ± 4.5	12.8 ± 5.5	0.018
Median	4.9	8.8	11.8	
Range	1.0–13.4	4.3–19.9	5.8–19.9	
PSAD (ng/ml/ml)				
Mean ± SD	0.12 ± 0.05	0.25 ± 0.13	0.33 ± 0.14	0.002
Median	0.11	0.22	0.30	
Range	0.02–0.23	0.10–0.53	0.17–0.53	
SelectMDx score, *n* (%)				
Negative	32 (91.4)	1 (5.9)	0 (0)	0.002
Positive	3 (8.6)	16 (94.1)	7 (100)	
Probability for csPCa (%)				
Mean ± SD	1.5 ± 5.4	33.6 ± 21.3	49.4 ± 16.6	
Median (range)	0( 0–26)	30.0 (0–81)	52.0 (30–81)	
mpMRI PI-RADS score, *n* (%)				
PI-RADS 1–2	28 (80.0)	1 (5.9)	0 (0)	0.001
PI-RADS 3	5 (14.3)	6 (35.3)	1 (14.3)	
PI-RADS 4–5	2 (5.7)	10 (58.8)	6 (85.7)	
SelectMDx score and mpMRI PI-RADS score, *n* (%)				
SelectMDx positive, mpMRI positive	3 (8.6)	15 (88.2)	7 (100)	0.001
SelectMDx negative, mpMRI negative	28 (80.0)	0 (0)	0 (0)	
SelectMDx positive, mpMRI negative	0 (0)	1 (5.9)	0 (0)	
SelectMDx negative, mpMRI positive	4 (11.4)	1 (5.9)	0 (0)	
PSA and mpMRI PI-RADS score, *n* (%)				
PSA ≥ 3 ng/ml, mpMRI positive	5 (14.3)	16 (94.1)	7 (100)	0.001
PSA < 3 ng/ml, mpMRI negative	2 (5.7)	0 (0)	0 (0)	
PSA ≥ 3 ng/ml, mpMRI negative	26 (74.3)	1 (5.9)	0 (0)	
PSA < 3 ng/ml, mpMRI positive	2 (5.7)	0 (0)	0 (0)	

*n* number, *SD* standard deviation, *PSA* prostate-specific antigen, *PSAD* PSA density, *mpMRI* multiparametric magnetic resonance imaging, *PI-RADS* prostate imaging reporting and data system, *PCa* prostate cancer, *csPCa* clinically significant PCa

### Predictive value of SelectMDx for PCa and csPCa at biopsy

The performance of SelectMDx compared to that of total PSA and mpMRI PI-RADS score to predict PCa and csPCa at biopsy is reported in Table 3. SelectMDx showed slightly lower sensitivity (94.1 versus 100.0%) but higher specificity (91.4%) than total PSA (17.1%) and the same sensitivity but higher specificity than mpMRI PI-RADS (80.0%) in predicting PCa positivity at biopsy (Table 3). The association of SelectMDx in combination with mpMRI rather than PSAD plus mpMRI showed higher specificity (both 91.4%) in comparison with the association of PSA plus mpMRI (85.7%). In terms of csPCa predictive value, again SelectMDx showed higher specificity (73.3%) than PSA (13.3%) and mpMRI PI-RADS (64.4%) as the association of SelectMDx plus mpMRI (75.6%) versus PSA plus mpMRI (68.9%); the association of PSAD plus mpMRI showed the highest specificity of 80.0% (Table 3). Visual comparison of overall diagnostic accuracy between each diagnostic tool alone or in combination have been summarized and graphically displayed as AUC in Supplementary Figure 2a and 2b.

**Table 3:** Performance of individual parameters and their combination to predict PCa and csPCa on biopsy (% and 95% CI)

Parameter	All PCa				csPCa			
	Sensitivity	Specificity	NPV	PPV	Sensitivity	Specificity	NPV	PPV
Total PSA	100.0 (0.779–1.000)	17.1 (0.078–0.332)	100.0 (1.000–1.000)	37.0 (0.230–0.509)	100.0 (0.590–1.000)	13.3 (0.060–0.267)	100.0 (1.000–1.000)	15.2 (0.048–0.256)
SelectMDx	94.1 (0.707–1.000)	91.4 (0.767–0.977)	97.0 (0.911–1.000)	84.2 (0.678–1.000)	100.0 (0.590–1.000)	73.3 (0.588–0.841)	100.0 (1.000–1.000)	36.8 (0.152–0.585)
mpMRI PI-RADS	94.1 (0.707–1.000)	80.0 (0.637–0.901)	96.6 (0.899–1.000)	69.6 (0.508–0.884)	100.0 (0.590–1.000)	64.4 (0.498–0.768)	100.0 (1.000–1.000)	30.4 (0.116–0.492)
SelectMDx + mpMRI PI-RADS	88.2 (0.642–0.977)	91.4 (0.767–0.977)	94.1 (0.862–1.000)	83.3 (0.661–1.000)	100.0 (0.590–1.000)	75.6 (0.611–0.858)	100.0 (1.000–1.000)	38.9 (0.164–0.614)
Total PSA + mpMRI PI-RADS	94.1 (0.707–1.000)	85.7 (0.700–0.941)	96.8 (0.906–1.000)	76.2 (0.580–0.944)	100.0 (0.590–1.000)	68.9 (0.542–0.805)	100.0 (1.000–1.000)	33.3 (0.132–0.535)
PSAD + mpMRI PI-RADS	76.5 (0.521–0.908)	91.4 (0.767–0.977)	88.9 (0.786–0.992)	81.3 (0.621–1.000)	100.0 (0.590–1.000)	80.0 (0.659–0.892)	100.0 (1.000–1.000)	43.8 (0.194–0.681)

*PCa* prostate cancer, *csPCa* clinically significant prostate cancer, *CI* confidence interval, *PSA* prostate-specific antigen, *mpMRI* multiparametric magnetic resonance imaging, *PI-RADS* prostate imaging reporting and data system, *NPV* negative predictive value, *PPV* positive predictive value, *PSAD* PSA density

**Fig. 3:**
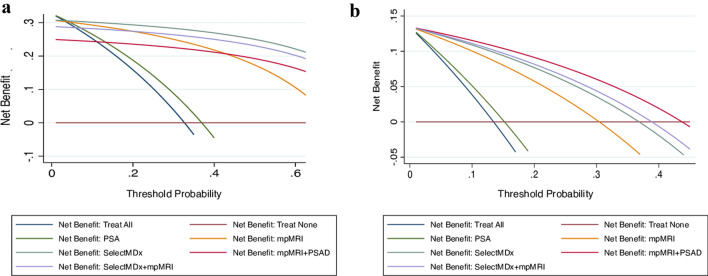
Decision curve analysis comparing clinical utility of SelectMDx score, mpMRI, total PSA, and the associations SelectMDx + mpMRI, and PSAD + mpMRI for detecting PCa (**a**) and csPCa (**b**)

Additionally, DCA curves were performed to explore the clinical net benefit of both tests alone or in combination, according to the different aforementioned strategies. While DCA for SelectMDx alone revealed the highest net benefit for the detection of PCa, the combination of mpMRI and PSAD was found to be the best strategy for the detection of csPCa albeit only slightly superior to SelectMDx and mpMRI combination (Fig. 3a, b).

### Association of SelectMDx score with mpMRI PI-RADS score

The distribution of SelectMDx scores on the basis of PI-RADS results at mpMRI is reported in Supplementary Figure 2b and compared to PSA distribution (Supplementary Figure 2a). SelectMDx positivity significantly increased according to PI-RADS score from 3.4% in PI-RADS 1–2 to 72.7 and 83.3% in PI-RADS 3 and 4–5, respectively, (*p* < 0.0001) as well as PSA values were differently distributed among PI-RADS score categories (*p* = 0.02) (Fig. 1b and Supplementary File 2b).

To investigate the potential added value of implementing SelectMDx in the mpMRI diagnostic pathway, we analyzed cases with discordant tests. Among PI-RADS 1–2 cases, 1 out of 29 (3.4%) patients would undergo biopsy according to SelectMDx results, with the detection of the only PCa present in this category. Sparing biopsy in patients with a PI-RADS score 4–5 and a negative SelecMDx test (*n* = 2/12), would result in 2 (16.7%) avoided biopsies within this category, without missing any PCa. Among PI-RADS score 3 cases, performing prostate biopsy only in those with a positive SelectMDx, would result in the detection of 5/6 (83.3%) PCa, while avoiding biopsy in those with a negative SelectMDx would miss 1/6 (16.7%) PCa diagnosed within this category, without missing any csPCa.

Performing prostate biopsy in patients with a PI-RADS score 1–2 and PSAD ≥ 0.15, would result in 8 (27.6%) biopsies performed in this category (without detecting any PCa). Sparing biopsy in patients with a PI-RADS score 4–5 and PSAD < 0.15, would result in 1 (8.3%) avoided biopsies within this category, without missing any PCa. Among PI-RADS score 3 cases, performing prostate biopsy only in those with PSAD ≥ 0.15, would result in the detection of 3/6 (50.0%) PCa, while avoiding biopsy in those with PSAD < 0.15 would miss 3/6 (50.0%) PCa diagnosed within this category, without missing any csPCa.

## Discussion

In order to reduce unnecessary prostate biopsies and diagnosis of insignificant PCa, a number of tests have been proposed to improve PSA performance [17, 18]. Leyten et al. presented a 3-gene urinary panel using HOXC6, TRD1 and DLX1 [10]. Van Neste et al. found that HOXC6 and DLX1 were sufficient for prediction of positive prostate biopsy and csPCa with a sensitivity of 91%, specificity of 36% and NPV of 93% [12]. At now only few clinical trials prospectively investigated SelectMDx in patients with an initial suspicious for PCa and European Urology Association (EAU) Guidelines did not recommend its routine use [2, 19].

Recently Haese et al. in a multi-center study on men with a PSA level < 10 ng/ml showed an AUC of 0.82, 89% sensitivity and 53% specificity in predicting csPCa at biopsy [19]. Pepe et al., analyzing a series of PCa patients managed with active surveillance, found a diagnostic accuracy of 70.3% for SelectMDx compared to 84.5% for mpMRI in the detection of csPCa, underlining that the performance of SelectMDx might improve in case of initial or repeat biopsy [20]. Roumiguie et al. evaluated SelectMDx in a cohort of upfront mpMRI and image-guided biopsy patients and reported a similar accuracy between these tools (AUC = 0.67 for both). In their series complementing PI-RADS score with either SelecMDx or the sole clinical variables resulted in a higher performance compared to either test separately (AUC = 0.73 and 0.80, respectively) [21].

In our prospective study on men selected for initial prostate biopsy on the basis of PSA values or DRE results, SelectMDx score was positive in 94.1 and 100% of PCa and csPCa, respectively, and in only 8.6% of cases with no PCa at biopsy. SelectMDx showed similar sensitivity but higher specificity than total PSA and mpMRI PI-RADS in predicting both PCa and csPCa at biopsy. The association of SelectMDx plus mpMRI showed higher specificity than the association of PSA plus mpMRI, and comparable to that of PSAD plus mpMRI in predicting PCa diagnosis at biopsy. For the detection of csPCa the association of PSAD plus mpMRI resulted in the best performance. Moreover, SelectMDx showed a significant association with mpMRI results in terms of PI-RADS score. SelectMDx positivity significantly increased according to PI-RADS score (*p* < 0.0001). At the same time PSA distribution according to PI-RADS scores reached statistically significant difference among the different categories (*p* = 0.02).

Regarding PI-RADS 3 cases, which are equivocal by nature, SelectMDx might aid the decision making scenario when to perform prostate biopsy versus observation [22]. In our experience performing biopsy only in those with a positive SelectMDx, would result in the detection of 83.3% PCa, while using PSAD ≥ 0.15 to decide on the need for biopsy would result in a lower PCa detection rate (50%), yet missing any csPCa with both strategies.

In conclusion, our analysis suggests several points of interest: (1) SelectMDx revealed to be a reliable and valid diagnostic tool for detection of PCa with diagnostic performance comparable if not superior than commonly adopted tools. (2) We were not able to confirm what previously demonstrated by other experiences with regard of the diagnostic accuracy for the detection of clinically significant disease. Nevertheless, at DCA the associations SelctMDx + mpMRI and PSAD + mpMRI appeared to be almost comparable or at least non-significantly inferior for the net benefit strategy with regard to the sole PCa outcome. Additionally, we would be cautious speculating on the implementation of the combination strategy SelectMDx + mpMRI for the detection of csPCa, since PSAD + mpMRI revealed better reliability for this specific aim of interest.

Our study warrants certain limitations. First, the number of our population and of csPCa diagnosed are limited. Our preliminary results with the implementation of SelectMDx might on one hand be defined as highly performant with regard of overall PCa detection but on the other, their real impact on clinical practice especially for identification of csPCa should not be considered as definitive as revealed from the present series, but would certainty deserve further investigation in larger, prospective and possibly multi-center studies. Moreover, even if recent studies showed SelectMDx quality-adjusted life years (QALYs) might increase while saving healthcare costs in the initial diagnosis of PCa, making the use of SelectMDx prior to biopsy a potentially cost-effective strategy compared to the standard of care seems up to now, distant from clinical reality due to lacking of high level of evidence justifying such approach [23–25]. A simulation study comparing the performance of mpMRI and biomarkers, revealed that the former would be the optimal strategy as it minimizes cost and maximizes effectiveness [25]. However, these results are sensitive to the ability in detecting insignificant PCa, and, more importantly, there is still uncertainty regarding the interplay of these tests. Thus, the cost-effectiveness of SelectMDx compared with that of mpMRI is an important aspect to address in future research in order to guide clinicians in this area.

## Conclusion

In our personal clinical experience SelectMDx demonstrated to be a simple method to aid clinicians in PCa diagnosis whereas elevated costs represented the main limit to be routinely implemented for an extended population. The real impact on clinical practice, especially for identification of cases suffering from clinically significant disease, would certainty deserve further investigation in larger, prospective and possibly multi-center studies.

## Electronic supplementary material

Below is the link to the electronic supplementary material.

**Supplementary Figure 1:** (a) Total PSA distribution (mean, median, Standard Deviation (SD), range) in negative, PCa, and csPCa cases at biopsy; (b) Total PSA distribution (mean, median, Standard Deviation (SD), range) according to PI-RADS score at mpMRI (TIFF 17589 kb)

**Supplementary Figure 2: (a)** SelectMDx score *versus* total PSA and mpMRI PI-RADS score performance evaluated as area under the curve (AUC) of the receiver operating characteristics (ROC) in predicting PCa at biopsy; **(b)** SelectMDx + mpMRI PI-RADS score *versus* PSAD + mpMRI PI-RADS score performance evaluated as area under the curve (AUC) of the receiver operating characteristics (ROC) in predicting PCa at biopsy (TIFF 17589 kb)

## References

[1] Sciarra A, Gentilucci A, Salciccia S (2018). Psychological and functional impact of different primary treatments for prostate cancer: a comparative prospective analysis. Urol Oncol.

[2] Mottet N, van den Bergh RCN, Briers E, Cornford P, De Santis M, Fanti S et al (2019) EAU-ESTRO-SIOG Guidelines on Prostate Cancer. https://uroweb.org/guidelines/prostate-cancer

[3] Draisma G, Etzioni R, Tsodikov A, Mariotto A, Wever E, Gulati R (2009). Lead time and overdiagnosis in prostate-specific antigen screening: importance of methods and context. J Natl Cancer Inst.

[4] Panebianco V, Barchetti F, Sciarra A, Ciardi A, Indino EL, Papalia R (2015). Multiparametric magnetic resonance imaging vs. standard care in men being evaluated for prostate cancer: a randomized study. Urol Oncol.

[5] Kasivisvanathan V, Rannikko AS, Borghi M, Panebianco V, Mynderse LA, Vaarala MH (2018). MRI-targeted or standard biopsy for prostate-cancer diagnosis. N Engl J Med.

[6] Nordström T, Vickers A, Assel M, Lilja H, Grönberg H, Eklund M (2015). Comparison between the four-kallikrein panel and prostate health index for predicting prostate cancer. Eur Urol.

[7] Groskopf J, Aubin SM, Deras IL, Blase A, Bodrug S, Clark C (2006). APTIMA PCA3 molecular urine test: development of a method to aid in the diagnosis of prostate cancer. Clin Chem.

[8] Gittelman MC, Hertzman B, Bailen J, Williams T, Koziol I, Henderson RJ (2013). PCA3 molecular urine test as a predictor of repeat prostate biopsy outcome in men with previous negative biopsies: a prospective multicenter clinical study. J Urol.

[9] Cucchiara V, Cooperberg MR, Dall’Era M, Lin DW, Montorsi F, Schalken JA (2018). Genomic markers in prostate cancer decision making. Eur Urol.

[10] Leyten GH, Hessels D, Smit FP, Jannink SA, de Jong H, Melchers WJ (2015). Identification of a candidate gene panel for the early diagnosis of prostate cancer. Clin Cancer Res.

[11] Hendriks RJ, van der Leest MMG, Dijkstra S, Barentsz JO, Van Criekinge W, Hulsbergen-van de Kaa CA (2017). A urinary biomarker-based risk score correlates with multiparametric MRI for prostate cancer detection. Prostate.

[12] Van Neste L, Hendriks RJ, Dijkstra S, Trooskens G, Cornel EB, Jannink SA (2016). Detection of high-grade prostate cancer using a urinary molecular biomarker-based risk score. Eur Urol.

[13] Weinreb JC, Barentsz JO, Choyke PL, Cornud F, Haider MA, Macura KJ (2016). PI-RADS prostate imaging—reporting and data system: 2015, version 2. Eur Urol.

[14] Epstein JI, Egevad L, Amin MB, Delahunt B, Srigley JR, Humphrey PA (2016). The 2014 international society of urological pathology (ISUP) consensus conference on gleason grading of prostatic carcinoma: definition of grading patterns and proposal for a new grading system. Am J Surg Pathol.

[15] Hugosson J, Roobol MJ, Månsson M, Tammela TLJ, Zappa M, Nelen V (2019). A 16-yr follow-up of the European randomized study of screening for prostate cancer. Eur Urol.

[16] Andriole GL, Crawford ED, Grubb RL, Buys SS, Chia D, Church TR (2012). Prostate cancer screening in the randomized prostate, lung, colorectal, and ovarian cancer screening trial: mortality results after 13 years of follow-up. J Natl Cancer Inst.

[17] Carlsson SV, Roobol MJ (2017). Improving the evaluation and diagnosis of clinically significant prostate cancer in 2017. Curr Opin Urol.

[18] Fujita K, Nonomura N (2018). Urinary biomarkers of prostate cancer. Int J Urol.

[19] Haese A, Trooskens G, Steyaert S, Hessels D, Brawer M, Vlaeminck-Guillem V (2019). Multicenter optimization and validation of a 2-gene mRNA urine test for detection of clinically significant prostate cancer prior to initial prostate biopsy. J Urol.

[20] Pepe P, Dibenedetto G, Pepe L, Pennisi M (2020). Multiparametric MRI versus SelectMDx accuracy in the diagnosis of clinically significant PCa in men enrolled in active surveillance. Vivo.

[21] Roumiguié M, Ploussard G, Nogueira L, Bruguière E, Meyrignac O, Lesourd M (2020). Independent evaluation of the respective predictive values for high-grade prostate cancer of clinical information and RNA biomarkers after upfront MRI and image-guided biopsies. Cancers (Basel).

[22] Maggi M, Panebianco V, Mosca A (2020). Prostate imaging reporting and data system 3 category cases at multiparametric magnetic resonance for prostate cancer: a systematic review and meta-analysis. Eur Urol Focus.

[23] Dijkstra S, Govers TM, Hendriks RJ, Schalken JA, Van Criekinge W, Van Neste L (2017). Cost-effectiveness of a new urinary biomarker-based risk score compared to standard of care in prostate cancer diagnostics—a decision analytical model. BJU Int.

[24] Govers TM, Hessels D, Vlaeminck-Guillem V, Schmitz-Dräger BJ, Stief CG, Martinez-Ballesteros C (2019). Cost-effectiveness of SelectMDx for prostate cancer in four European countries: a comparative modeling study. Prostate Cancer Prostatic Dis.

[25] Sathianathen NJ, Kuntz KM, Alarid-Escudero F, Lawrentschuk NL, Bolton DM, Murphyet DG (2018). Incorporating biomarkers into the primary prostate biopsy setting: a cost-effectiveness analysis. J Urol.

