# Supplementary figures and images for: Prospective assessment of two-gene urinary test with multiparametric magnetic resonance imaging of the prostate for men undergoing primary prostate biopsy
Source: World J Urol. 2020 Jul 17;39(6):1869–77. doi: 10.1007/s00345-020-03359-w (PMC8217060; doi:10.1007/s00345-020-03359-w)

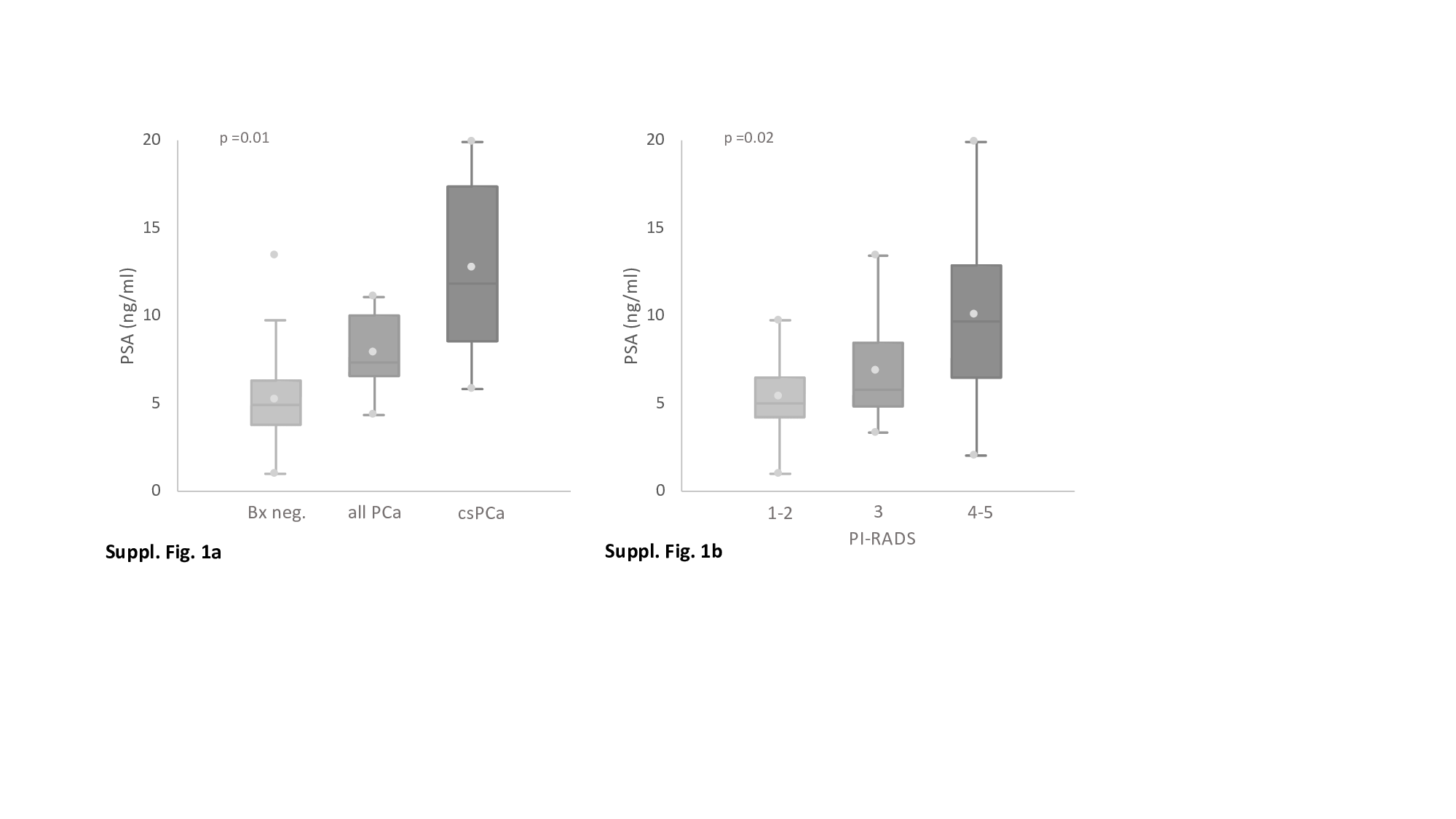

Supplement: Supplementary file 1 — Supplementary Figure 1: (a) Total PSA distribution (mean, median, Standard Deviation (SD), range) in negative, PCa, and csPCa cases at biopsy; (b) Total PSA distribution (mean, median, Standard Deviation (SD), range) according to PI-RADS score at mpMRI (TIFF 17589 kb) [file 345_2020_3359_MOESM1_ESM.tiff]

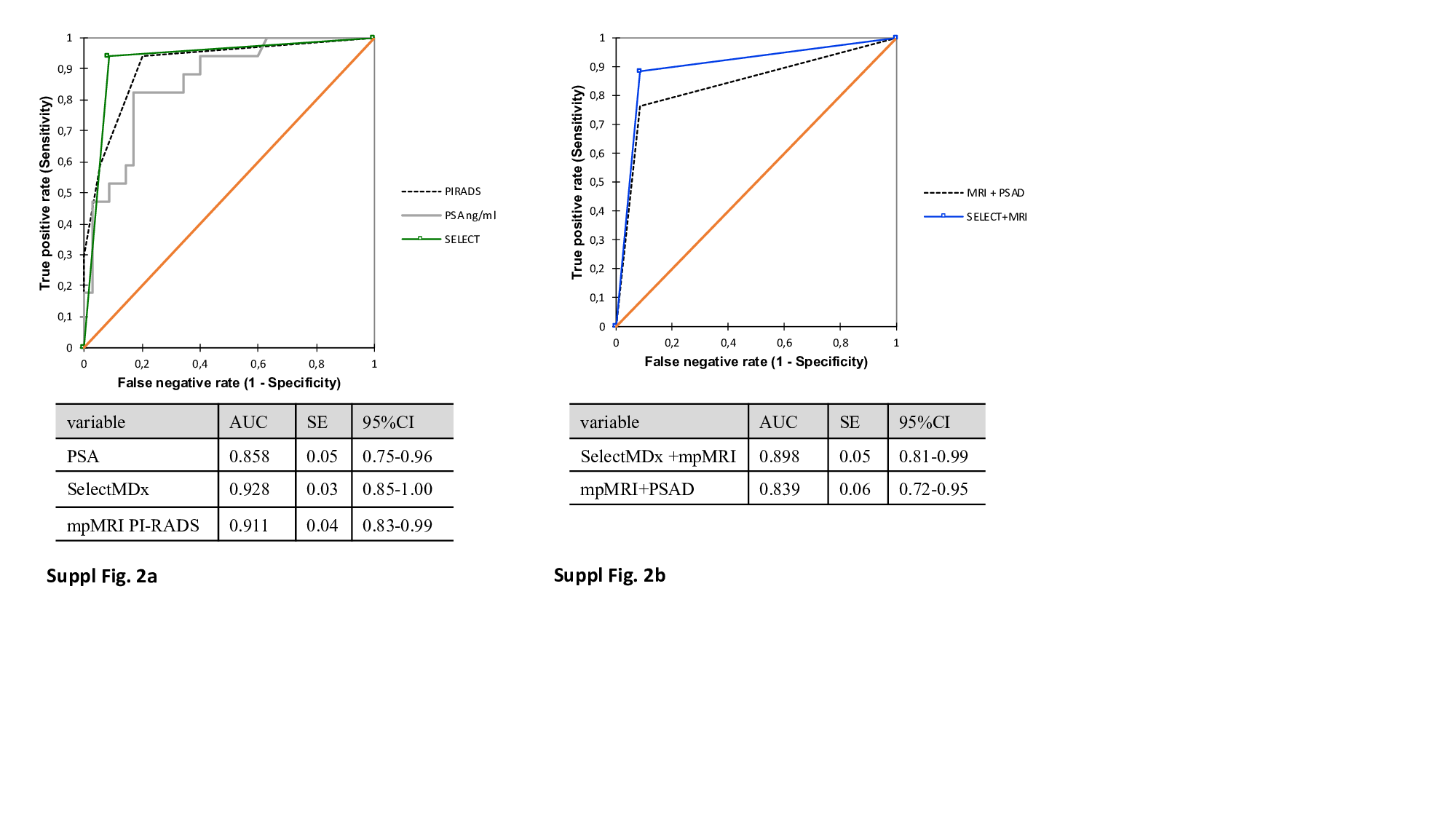

Supplement: Supplementary file 2 — Supplementary Figure 2: (a) SelectMDx score versus total PSA and mpMRI PI-RADS score performance evaluated as area under the curve (AUC) of the receiver operating characteristics (ROC) in predicting PCa at biopsy; (b) SelectMDx + mpMRI PI-RADS score versus PSAD + mpMRI PI-RADS score performance evaluated as area under the curve (AUC) of the receiver operating characteristics (ROC) in predicting PCa at biopsy (TIFF 17589 kb) [file 345_2020_3359_MOESM2_ESM.tiff]
